# Evaluating Sexual Dimorphism of the Muscle Spindles and Intrafusal Muscle Fibers in the Medial Gastrocnemius of Male and Female Rats

**DOI:** 10.3389/fnana.2021.734555

**Published:** 2021-10-01

**Authors:** Magdalena Gartych, Hanna Jackowiak, Dorota Bukowska, Jan Celichowski

**Affiliations:** ^1^Department of Neurobiology, Poznań University of Physical Education, Poznań, Poland; ^2^Department of Histology and Embryology, Poznań University of Life Sciences, Poznań, Poland

**Keywords:** sex differences, muscle spindles, motoneurons, density, morphometry

## Abstract

This study sought to investigate the sexual dimorphism of muscle spindles in rat medial gastrocnemius muscle. The muscles were cut transversely into 5–10 and 20 μm thick serial sections and the number, density, and morphometric properties of the muscle spindles were determined. There was no significant difference (*p* > 0.05) in the number of muscle spindles of male (14.45 ± 2.77) and female (15.00 ± 3.13) rats. Muscle mass was 38.89% higher in males (1.08 vs. 0.66 g in females), making the density of these receptors significantly higher (*p* < 0.01) in females (approximately one spindle per 51.14 mg muscle mass vs. one per 79.91 mg in males). There were no significant differences between the morphometric properties of intrafusal muscle fibers or muscle spindles in male and female rats (*p* > 0.05): 5.16 ± 2.43 and 5.37 ± 2.27 μm for male and female intrafusal muscle fiber diameter, respectively; 5.57 ± 2.20 and 5.60 ± 2.16 μm for male and female intrafusal muscle fiber number, respectively; 25.85 ± 10.04 and 25.30 ± 9.96 μm for male and female shorter muscle spindle diameter, respectively; and 48.99 ± 20.73 and 43.97 ± 16.96 μm for male and female longer muscle spindle diameter, respectively. These findings suggest that sexual dimorphism in the muscle spindles of rat medial gastrocnemius is limited to density, which contrasts previous findings reporting differences in extrafusal fibers diameter.

## Introduction

Muscle spindles are one of the most important proprioceptors in skeletal muscles, with afferent signals from these receptors functioning as excitatory input to motoneurons and enabling the control of body posture (Nielsen, 2002; Proske and Gandevia, 2009; Butler et al., 2017; Proske, 2019). The spindles are comprised of a bundle of 2–10 intrafusal muscle fibers aligned parallel to external muscle fibers and covered by a fusiform connective tissue capsule (Fitz-Ritson, 1982). The spindles are innervated by Ia and II afferent fibers and receive efferent innervation from γ and β motoneurons (Fitz-Ritson, 1982; Muramatsu et al., 2017; McComas et al., 2018). The afferent endings of muscle spindles are excited by muscle stretching and/or intrafusal muscle fiber contraction.

The medial gastrocnemius is a hindlimb muscle that is strongly activated during rapid movement and is essential for locomotion. This muscle type is expected to have a low muscle spindle density (Al-Mallak, 2006), while muscles with high muscle spindle density are generally involved in fine movements (Fitz-Ritson, 1982). Muscle spindle density and distribution vary across muscle type and mass (Kokkorogiannis, 2004; Banks, 2006; Zvaritch and MacLennan, 2015), different mammalian species (Scott and Young, 1987; Bredman et al., 1991; Lionikas et al., 2013), and age (Milburn, 1973; Winarakwong et al., 2004; Kim et al., 2007). Various methods can be used to determine the number and dimensions of the muscle spindles in muscle cross-sections (Patten and Ovalle, 1992; Eldred et al., 1998; Kierner et al., 1999), with the most efficient being Masson-Goldner trichrome dyeing for the visualization of connective tissue (Boyd-Clark et al., 2002).

Although several studies have investigated differences in the number and density of muscle spindles in rat skeletal muscles, the majority of this work involved only males (Kim et al., 2007; Elsohemy et al., 2009; Muramatsu et al., 2017) or did not distinguish between genders (Pang et al., 2014). To date, there has been no systematic investigation into potential differences in rat muscle spindle number, density, or morphometric properties across sexes. The muscle mass and the proportion of different muscle fiber types in rats are known to be dependent on the level of male hormones present during ontogeny (Bell, 2018). Recent studies have reported considerable sex-dependent differences in the muscle mass and diameter of extrafusal muscle fibers in rat medial gastrocnemius, suggesting differences in the morphometric properties of both intrafusal muscle fibers and muscle spindles (Mierzejewska-Krzyżowska et al., 2011; May et al., 2018). On the other hand, the staining of motoneurons in the motor nucleus of the medial gastrocnemius revealed that male rats had a greater number of α-motoneurons than females, but a similar number of γ-motoneurons (Mierzejewska-Krzyżowska et al., 2014). This suggests that the number of muscle spindles in the medial gastrocnemius of male and female rats is similar and, therefore, the density of these receptors must be different. Building off these findings, this study sought to determine whether sexual dimorphism could be observed in rat medial gastrocnemius muscle by measuring the number, density, and morphometric properties of the muscle spindles and intrafusal muscle fibers.

## Materials and Methods

### Animals and Muscle Preparation

Experiments were performed on six male and six female 3-month-old Wistar rats. The mean body mass of the male and female rats was 351.00 ± 14.45 and 195.75 ± 10.44 g, respectively (*p* ≤ 0.0001). Animals were maintained in standard laboratory cages (two rats/cage; genders isolated) in a temperature- and humidity-controlled (22 ± 2°C and 55 ± 10%, respectively) animal house under a 12 h light/dark cycle. The rats were fed a standard laboratory diet *ad libitum* (Labofeed B, Poland) and had free access to tap water. Experiments were performed in accordance with the European Union and Polish Law on Animal Protection and approved by the Local Bioethics Committee (Permission Number: 63/2017). All experimental protocols employed the 3Rs principles to minimize suffering and limit the number of animals used.

Animals were euthanized with an overdose of sodium pentobarbital (Morbital, 180 mg/kg, i.p.). The skin and part of the external muscles were removed from the left and right hind limbs of the animals. The preparations having the medial gastrocnemius muscles in the neutral length and attached to the foot and femur were preserved in 4% formalin solution for 7 days to fix the muscles in their normal shape. The fixed muscles were excised and weighed (without the Achilles tendon), giving a mean muscle mass of 1.08 ± 0.08 and 0.66 ± 0.07 g for male and female rats, respectively (*p* ≤ 0.0001). The muscles were then reimmersed in 4% formalin solution.

### Staining Procedure

After 3–5 days fixation in 10% neutral formalin, the muscle samples were dehydrated in an ethanol series (50–96%) and methyl benzoate and embedded in paraffin. The muscles were then cut transversely from the proximal to distal muscle attachment using a rotatory microtome Leica RM 2055 to give 5–10 and 20 μm thick serial sections. Histoslides were then stained using the Masson-Goldner trichome method to calculate the number of muscle spindles and determine/estimate their precise distribution in the muscles. Briefly, histological slices were first deparaffinized in xylene I–III for 5 min each, rehydrated in a descending alcohol series (2 × 96% alcohol for 3 min, 90% alcohol for 3 min, 80% alcohol for 3 min, and 70% alcohol for 3 min), and washed in distilled water for 5 min. The slices were then stained in Mayer’s hematoxylin solution for 10–15 min, rinsed with warm tap water for 10 min, and washed in distilled water for 10 min. Sections were then stained in acidic-fuchsin solution for 10 min and washed in distilled water. The sections were differentiated in phosphomolybdic acid solution for 3 min, stained in aniline blue solution for 10 min, briefly rinsed in distilled water, and differentiated in 1% acetic acid solution for 1 min. The stained sections were dehydrated in ascending ethanol (washed in 70, 80% for 30 s, 90% for 5 min, and 2× 96% for 5 min) and toluene (I–III line for 5 min each) series and mounted using DPX medium.

### Muscle Spindle Measurements

The number and distribution of muscle spindles in the muscles were determined by light microscopy (Prolab, Poland). Morphometric analysis photographs of each muscle spindle were obtained on a Nikon Optihot-2 light microscope fitted with a Nikon DS-Fi1c camera (Tokyo, Japan), using 10× (0.38 μm/pixel), 40× (0.10 μm/pixel), and 63× (0.04 μm/pixel) zoom lenses. The morphometric traits of the spindles were measured in the equatorial muscle region using NIS-Elements Basic Research software (Nikon). The equatorial region was deemed a half-length of the muscle spindle. The muscle spindle diameter was calculated as the mean shorter and longer diameters. Only the shorter diameter of the intrafusal muscle fibers was used for comparing sex-related differences.

### Statistical Analysis

Data are reported as the mean ± SD. Statistical analysis was performed using Statistica v.13.0 software (TIBCO Software Inc., Tulsa, OK, United States). Significant differences between the sexes were evaluated using a Student’s *t*-test for normally distributed data and also Student’s *t*-test with independent variance estimation or a Mann–Whitney U test when a normal distribution could not be confirmed, with *p* < 0.05 accepted as statistically significant.

## Results

**FIGURE 1:**
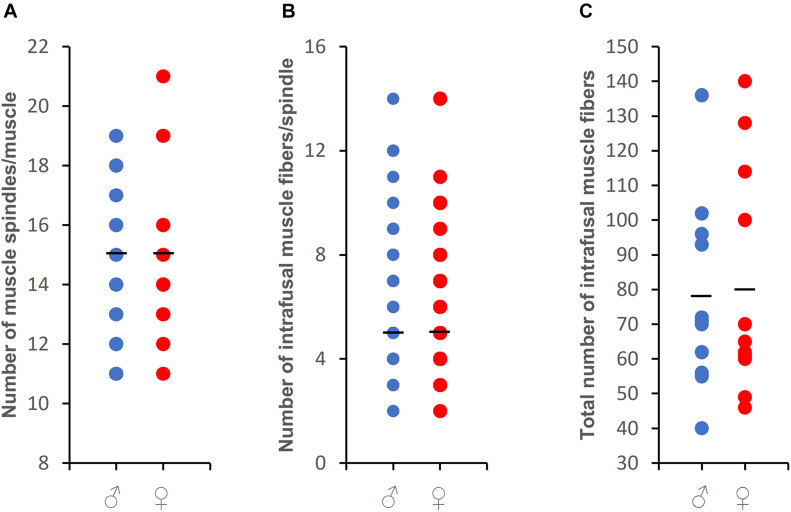
The number of muscle spindles found for each of studied muscles **(A)**, the number of intrafusal muscle fibers per spindle **(B)**, and the total number of intrafusal muscle fibers for each muscle (a sum of all fibers in each of studied muscle spindles) **(C)** for the medial gastrocnemius in male and female rats. Note that data some data are represented by overlapping symbols. A short vertical bar represent the mean value.

The body mass, muscle mass, number, density, and morphometry of medial gastrocnemius muscle spindles were obtained for both male and female Wistar rats (Table 1). Male rats had 44.2% greater body mass and 38.9% greater muscle mass than females. A total of 159 (11–19 receptors/muscle) and 180 (11–21 receptors/muscle) muscle spindles were identified across all examined male and female muscles, respectively. While the mean number of muscle spindles/muscle did not differ between males and females (Figure 1A), the density of spindles appeared to be different. In general, one muscle spindle was present per 79.91 and 51.14 mg of muscle mass in males and females, respectively (*p* < 0.01).

**TABLE 1:** Mean ± SD and range of body mass, muscle mass, number and density of muscle spindles, and morphometric properties of muscle spindles and intrafusal muscle fibers for male (♂) and female (♀) rats.

Sex	Body mass^a^ (g)	Muscle mass^a^ (g)	Number of muscle spindles^a^	Muscle spindle density^b^ (mg)	Number of intrafusal fibers/spindle^c^	Shorter diameter of intrafusal fibers^c^ (μm)	Shorter diameter of muscle spindles^c^ (μm)	Longer diameter of muscle spindles^c^ (μm)	The total number of intrafusal muscle fibers per muscle^a^
♂	351.00 ± 14.45 232–366	1.08 ± 0.08 0.95–1.20	14.45 ± 2.77 11–19	79.91 ± 15.56 59.74–92.31	5.57 ± 2.20 2–14	5.16 ± 2.43 0.67–14.09	25.85 ± 10.14 9.15–55.69	48.99 ± 20.73 17.71–112.85	77.55 ± 27.11 40–136
♀	195.75 ± 10.44 185–207	0.66 ± 0.07 0.57–0.74	15.00 ± 3.13 11–21	51.14 ± 9.08 40.71–66.36	5.60 ± 2.16 2–14	5.37 ± 2.27 1.33–14.44	25.30 ± 9.96 9.08–56.68	43.97 ± 16.96 15.29–109.78	79.67 ± 32.12 46–140
*p*	**	**	NS	*	NS	NS	NS	NS	NS

*Significant differences across sexes were analyzed using a ^*a*^Student’s *t*-test, ^*b*^Student’s *t*-test with independent variance estimation, or ^*c*^Mann–Whitney U test.*

NS, non-significant (*p* > 0.05), *p < 0.01, and **p < 0.001.

**FIGURE 2:**
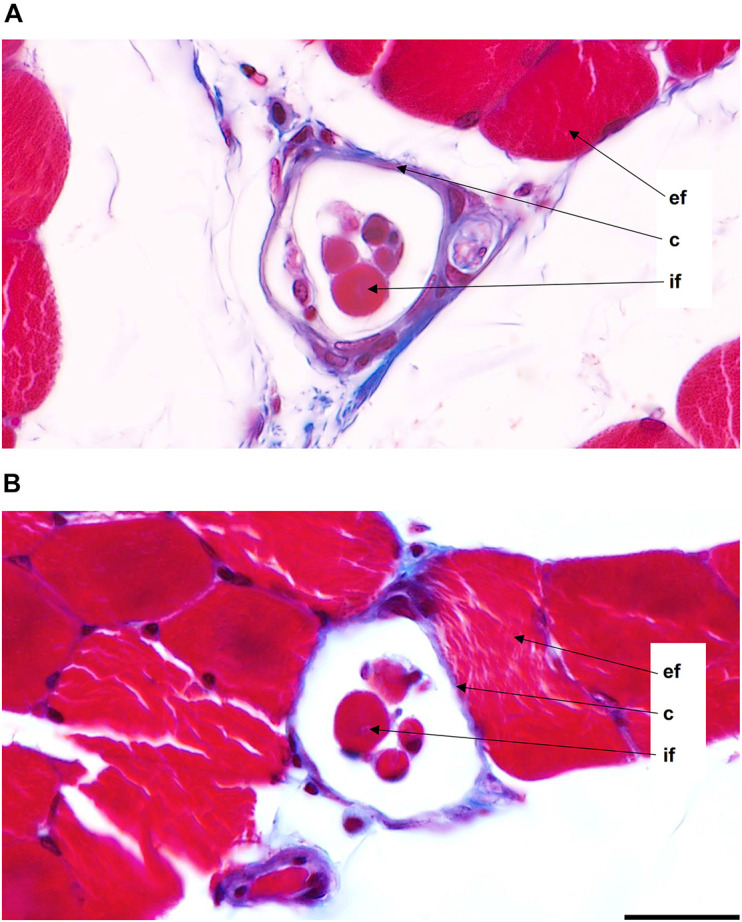
Muscle spindles from the middle region of the medial gastrocnemius muscles of male **(A)** and female **(B)** 3-month-old Wistar rats. Images were acquired at 63× magnification (0.04 μm/pixel). ef, extrafusal muscle fibers; c, capsule; if, intrafusal muscle fibers. Scale bar: 20 μm.

Male and female muscle transversal sections containing muscle spindles were imaged (Figure 2). The morphometric properties of muscle spindles and intrafusal muscle fibers were compared between male and female muscles (a total of 825 and 968 intrafusal muscle fibers, respectively; Table 1). The intrafusal muscle fiber diameter and longer diameter of the muscle spindles were slightly greater in males (3.91 and 10.25%, respectively) than in females. However, there were no significant differences between intrafusal muscle fiber diameter, or the longer and shorter diameters of the muscle spindles in male and female muscles as well as the number of intrafusal muscle fibers per one receptor (Figure 1B). Notably, both male and female muscles had a range of 2–14 fibers/muscle spindle. Moreover, the total numbers of intrafusal muscle fibers in individual male and female muscles calculated as a sum of all intrafusal fibers in all identified muscle spindles in a given muscle were not different (Figure 1C).

## Discussion

There are limited studies characterizing sex-related differences in the muscle spindles of skeletal muscles. The sole report that considered sex investigated extraocular muscles and found significant differences between the total number of muscle spindles in male and female humans (269 vs. 217.5, respectively) (Lukas et al., 1994). The present study provides the first systematic investigation of sexual dimorphism in the muscle spindles of hindlimb skeletal muscle, focusing specifically on quantity, density, and morphometric properties. The number of muscle spindles in the muscles of male and female rats was similar; however, the density of these receptors was lower in males. This observation aligns with previous work, which found the number of γ-motoneurons innervating intrafusal muscle fibers to be similar between male and female rats (Mierzejewska-Krzyżowska et al., 2014). There were no sex-related differences in the number and diameter of intrafusal muscle fibers in muscle spindles, which contrasts a previous report that the extrafusal muscle diameter was 14% greater in males (Mierzejewska-Krzyżowska et al., 2011). Additionally, the total number of intrafusal muscle fibers in male and female muscles was similar, contrasting to about 1.5 times higher number of extrafusal muscle fibers in males (Mierzejewska-Krzyżowska et al., 2011). Male hormones influence the diameter of muscle fibers and play a major role in muscle mass (Eason et al., 2000; English and Schwartz, 2002). Therefore, the finding that intrafusal muscle fiber diameter is similar between the sexes suggests that male hormones have a limited influence on intrafusal muscle fibers. Importantly, Österlund et al. (2011) reported that muscle spindles reach maturity before the extrafusal fiber population in human masseter and biceps brachii muscles. In addition, the number of muscle spindles, γ-motoneurons innervating intrafusal muscle fibers, and intrafusal muscle fibers/receptor are similar in males and females. Based on these observations, it is likely that the motor innervation of muscle spindles and the number of muscle spindles and intrafusal muscle innervated by individual γ-motoneurons are not sexually dimorphic. This is in contrast to previously reported sex differences in the motor unit innervation ratio (the number of extrafusal muscle fibers innervated by one α-motoneuron), which was 26% higher in male rat medial gastrocnemius muscle (Mierzejewska-Krzyżowska et al., 2011).

The number of muscle spindles identified in rat medial gastrocnemius was consistent with previous work performed using male rats. Kim et al. (2007) measured the number of muscle spindles in the medial gastrocnemius muscles of male Fischer rats and reported 11–12 and 11–15 spindles in rats aged 6.5 and 29.5 months, respectively. Muramatsu et al. (2017) reported 15.6 ± 2.3 and 16.3 ± 1.3 receptors in the medial gastrocnemius of 3-month-old and 5.5-month-old male Wistar rats, respectively. The average number of muscle spindles reported in 8-month-old male Fischer 344 rats was 16.7 ± 0.96 (Asano et al., 2019). Several studies have reported the number of muscle spindles in gastrocnemius muscles (including the medial and lateral head). Elsohemy et al. (2009) found 23.67 ± 2.52 spindles in adult Lewis rats. Pang et al. (2014) measured 26.7 ± 2.5 muscle spindles in 9-week-old Sprague-Dawley rats, although they did not distinguish between male and female. These observations additionally suggest that the two heads of the gastrocnemius muscle contain similar numbers of muscle spindles.

Several methods can be used to calculate the density of muscle spindles, the most popular being to divide the total number of spindles by the wet weight of the muscle. In this study, significant differences were observed in the muscle spindle density of male and female rats. The density of muscle spindles is likely relevant to the function of the skeletal muscles, particularly the ability to perform rapid or fine movements (Fitz-Ritson, 1982). Furthermore, the density of muscle spindles may differ for various muscles. For example, the density of muscle spindles in human longus colli and multifidus muscles were estimated to be 48.6 and 24.3/g, respectively (Boyd-Clark et al., 2002). Although this work was performed on ten men and six women, the authors did not analyze the data for sex differences. The muscle spindle density in the gastrocnemius muscle of rats reported in this work differs from that of Al-Mallak (2006), who found 5.78 muscle spindles per 1 g of muscle mass in adult rats. Banks (2006) systematically reviewed the density of muscle spindles in the gastrocnemius muscle of rats, cats, and humans and reported values of 40.3, 6.5, and 0.40 spindles per 1 g of muscle mass, respectively. Neither the Al-Mallak (2006) nor the Banks (2006) study indicated the sex of the animals.

The number of intrafusal muscle fibers varies greatly by species and muscle type; for example, 2–12 intrafusal muscle fibers are observed in human male extraocular muscle (Bruenech and Ruskell, 2001) while 1–16 are found in female bovine extraocular muscle (Maier, 2000). The number of fibers/muscle spindle measured in this study (2–14) is within this range and agrees with previous work investigating rat muscles. For example, the masticatory muscles of adult Sprague-Dawley rats of both sexes are reported to have 2–5 intrafusal muscle fibers (Lennartsson, 1980). Muramatsu et al. (2017) found 4.3 ± 0.8 and 4.3 ± 0.7 intrafusal muscle fibers in the medial gastrocnemius of 3-month-old and 5.5-month-old male rats, respectively.

No studies have investigated sex-related differences in the diameter of rat intrafusal muscle fibers. This work found the intrafusal fiber diameter in rat medial gastrocnemius muscles to be similar across the sexes (1–14 μm), which is within the range of diameters reported for mammalian muscles (e.g., 2–20 μm for human levator palpebrae superioris muscle) (Korzeniowska-Kromer and Wójtowicz-Kaczmarek, 2003). Muramatsu et al. (2017) reported the average cross-sectional area of intrafusal muscle fibers to be 41.5 ± 11.5 μm^2^ in 3-month-old male Wistar rats.

The longer diameter of the muscle spindles for males amounted to 48.99 ± 20.73 μm and for females (43.97 ± 16.96 μm). These measurements are consistent with the 42.29 ± 3.04 μm maximal diameter of muscle spindles in the gastrocnemius muscles of adult Lewis rats (Elsohemy et al., 2009). Pang et al. (2014) reported a 42.9 ± 5.5 μm diameter of muscle spindles in the medial gastrocnemius muscles of 9-week-old male and female Sprague-Dawley rats, while Sekiya et al. (1986) noted a range of 15–85 μm in Wistar rats 25–35 days after birth.

While male and female muscle spindles share similar morphometric properties, their density differs significantly. This observation could explain some functional differences associated with proprioceptive feedback from muscle receptors in humans. For example, women obtained better results than men in right-handed intra-modal matching tasks, suggesting greater congruency in visual and proprioceptive spaces (Sigmundsson et al., 2007). This could be associated with women’s more precise fine hand movement; e.g., more legible writing (Hartley, 1991). Interestingly, a study on the role of sex hormones in handwriting style found that women’s right-hand digit ratio correlated with the relative sexuality of their handwriting, but there was no corresponding relationship for men (Beech and Mackintosh, 2005). The lower density of muscle spindles in men might explain their decreased shoulder joint position sense acuity reported by Vafadar et al. (2015). Moreover, the muscle spindles in females probably have a better chance of functional recovery following the nerve damage as their receptors have higher density and their muscles are smaller (Elsohemy et al., 2009).

The study has some limitations concerning unexplored sex related differences in the studied muscle although additional experiments may answer these question. First, the structure of primary and secondary endings (of Ia and II sensory fibers) in muscle spindles was not analyzed. The quantity and distribution of Ia and II afferent sensory fibers may be defined with immunohistochemical markers like Parvalbumin or Runx3 and molecular markers specific to muscle spindles afferent sensory fibers like Heg and Inhbb (Wu et al., 2019). The identification of muscle fibers afferent subtypes may also be done with morphological methods based on confocal microscopy and also immunohistochemistry analysis (Sonner et al., 2017). Second, the distribution of muscle spindles and their relation to two compartments of medial gastrocnemius muscle are still unknown. The muscle compartments may be marked with the glycogen depletion technique (Taborowska et al., 2016). Third, the bag and chain intrafusal fibers in muscle spindles were not identified and differentiated.

## Conclusion

Overall, this study found that only the density of muscle spindles differed in male and female rat medial gastrocnemius, in contrast to previously reported differences in the number and diameter of extrafusal muscle fibers.

## Data Availability Statement

The original contributions presented in the study are included in the article/supplementary material, further inquiries can be directed to the corresponding author.

## Ethics Statement

The animal study was reviewed and approved by the Local Bioethics Committee in Poznań (Permission Number: 63/2017).

## Author Contributions

JC conceived and designed the research and conducted the data interpretation. MG wrote first draft of the manuscript. MG, HJ, DB, and JC collected and analyzed the data. HJ, DB, and JC reviewed the manuscript submitted for publication. All authors revised and approved the final version of the manuscript.

## Conflict of Interest

The authors declare that the research was conducted in the absence of any commercial or financial relationships that could be construed as a potential conflict of interest.

## Publisher’s Note

All claims expressed in this article are solely those of the authors and do not necessarily represent those of their affiliated organizations, or those of the publisher, the editors and the reviewers. Any product that may be evaluated in this article, or claim that may be made by its manufacturer, is not guaranteed or endorsed by the publisher.
